# Chiropractic students versus emergency care practitioners in simulated musculoskeletal emergencies

**DOI:** 10.4102/hsag.v30i0.3195

**Published:** 2025-10-31

**Authors:** Ivanna Balanco, Helen Slabber, Christopher Yelverton

**Affiliations:** 1 Department of Chiropractic, Faculty of Health Sciences, University of Johannesburg, Johannesburg, South Africa; 2 Department of Emergency Medical Care, Faculty of Health Sciences, University of Johannesburg, Johannesburg, South Africa

**Keywords:** chiropractic, diagnosis, emergency medical services, simulation, clinical competence, musculoskeletal disease

## Abstract

**Background:**

As primary contact practitioners, chiropractors and emergency care practitioners (ECPs) are first points of access for patients with musculoskeletal (MSK) complaints. A comparison of their diagnostic competency in distinguishing these presentations from underlying emergency pathologies remains an understudied area.

**Aim:**

To compare the diagnostic abilities of Master of Health Science (MHSc) chiropractic students and ECPs in distinguishing MSK from emergency conditions.

**Setting:**

The research was conducted at the University of Johannesburg, Faculty of Health Sciences, simulation laboratory.

**Methods:**

First-year (*n* = 10) and second-year Master’s (*n* = 10) chiropractic students and ECPs (*n* = 10) were assessed using standardised patient scenarios: meningitis, disc herniation and stroke, and assessed on diagnostic assessment, diagnosis and clinical and diagnostic investigation referrals.

**Results:**

Second-year MHSc students outperformed ECPs in the clinical management of a disc herniation case (Case 2; *p* < 0.01). Diagnostic accuracy was high (> 90%) for meningitis and stroke across all groups. Differences in investigation preferences emerged, with chiropractic students favouring advanced imaging and ECPs recommending more basic tests. No significant performance differences were found in the other two cases.

**Conclusion:**

Based on a simulated assessment, chiropractic students demonstrated equivalent competence to ECPs in diagnosing emergencies, but outperformed them in managing an MSK condition. These preliminary findings suggest chiropractors could contribute to the management of MSK burden in emergency departments.

**Contribution:**

MHSc chiropractic training enhances diagnostic proficiency in differentiating MSK disorders from emergent pathologies, an important competency for safe and effective practice as primary contact practitioners.

## Introduction

Musculoskeletal (MSK) conditions represent a significant global health burden, ranking as the second leading cause of disability worldwide and affecting approximately one in five working-age adults (Lowe, Taylor & Hill 2017; Weinstein 2016). Back pain, a prevalent MSK complaint, contributes substantially to reduced work productivity, absenteeism and healthcare costs (Ingram & Symmons 2018; Menke 2003). This high prevalence strains healthcare systems, leading to long wait times for specialist care, including surgery (Joshipura & Gosselin 2020). Internationally, MSK conditions account for approximately 13.8% – 25% of all emergency department (ED) visits, with low back pain being the most common complaint. In South Africa, the prevalence is comparably high or higher: non-traumatic MSK complaints are reported in 36% of primary care attendees, and trauma-related MSK injuries (from violence, road accident and gunshot wounds) constitute a large portion of ED presentations. The lifetime prevalence of low back pain in South Africa is as high as 62%, and trauma-related MSK injuries are a leading cause of ED visits (Fialho et al. 2011; Louw, Morris & Grimmer-Somers 2007; Parker & Jelsma 2010; Pasta et al. 2022; Taljaard, Maharaj & Hendrikse 2022).

Despite their frequent encounter with MSK complaints, many general practitioners and emergency care providers lack adequate training in MSK assessment and management (Denq et al. 2023; Suter et al. 2016; Woolf & Åkesson 2001). This shortfall may result in excessive imaging, unnecessary referrals and delayed care (Comer, Liang & Bishop 2014; Gagnon et al. 2022). Recent evidence also indicates that new graduates and emergency staff in emergency medical care (EMC) positions often feel unprepared to manage these conditions, underscoring the need for improved education (Denq et al. 2023).

Internationally, programmes have been established where physiotherapists have been included within ED to specifically address MSK conditions (Cassar et al. 2022; Harding et al. 2015; Matifat et al. 2023; Taylor et al. 2011). Chiropractors are well-positioned to fulfil a role of assisting in these environments, given their focused training in MSK diagnosis and management, including identifying serious pathologies mimicking MSK disorders (Haldeman et al. 2015; Murphy et al. 2011; University of Southern Denmark 2023). Studies suggest that integrating chiropractors into mainstream healthcare and potentially EDs, can reduce unnecessary imaging and referrals, lower costs and improve patient satisfaction (Kindermann, Hou & Miller 2014; Lisi et al. 2018).

This study addresses a critical gap in the literature by comparing the diagnostic acuity of chiropractors and emergency care practitioners (ECPs) in distinguishing non-urgent MSK disorders from emergent pathologies within a simulated environment. As primary contact clinicians, both groups are tasked with the initial assessment of potentially undifferentiated patients; however, their training pathways differ significantly. By benchmarking chiropractors against ECPs (a group specifically trained to recognise and triage medical emergencies), this research directly evaluates a core competency required for chiropractors to function safely in first-point-of-contact roles, such as ED settings, where the risk of misdiagnosing emergency pathologies which present similarly to MSK pathologies exists.

This study aimed to compare the diagnostic competencies of Master of Health Science (MHSc) chiropractic students in distinguishing MSK and emergency pathologies with those of ECPs.

### Study setting

The study was conducted in the University of Johannesburg, Faculty of Health Sciences, simulation laboratory, on the Doornfontein Campus.

## Research methods and design

### Study design

This was a quantitative, cross-sectional study, designed to be descriptive and comparative, to evaluate and compare the diagnostic proficiency of MHSc chiropractic students and ECPs. Specifically, the study assessed each group’s ability to accurately differentiate non-critical MSK conditions from non-MSK pathologies that constitute medical emergencies.

### Study populations and selection

The study population consisted of two distinct groups:

1. Master of Health Sciences chiropractic students: All students enrolled in the MHSc in chiropractic programme at the University of Johannesburg for the 2024 academic year. The total population size for first-year students was 36, and for second-year students, it was 41.
2. Emergency care practitioners: Qualified and currently practising practitioners holding a Bachelor of Health Sciences (BHS) degree in EMC.

A total of 30 participants were recruited through purposive sampling to ensure the inclusion of specific expertise and training backgrounds:

- Group 1 (Students): From the total student population (*N* = 77), a sample of 20 MHSc chiropractic students was selected. This included 10 first-year students (representing a 27.8% sampling fraction of the first-year cohort) and 10 second-year students (representing a 24.4% sampling fraction of the second-year cohort). This stratification allowed for the analysis of potential differences based on the level of postgraduate training.
- Group 2 (ECPs): Ten ECPs were recruited through professional networks. This group served as a benchmark of advanced diagnostic proficiency, as their specialised emergency care training is indicative of expert-level skills in identifying emergency pathologies. The sample size of 10 per group was chosen to enable a direct comparative analysis with the student subgroups while remaining logistically feasible.

### Inclusion criteria

For chiropractic students:

- Currently registered as a first- or second-year MHSc chiropractic student at the University of Johannesburg.
- Must have completed a 4-year undergraduate bachelor’s degree in chiropractic before enrolment in the Master’s programme.

For ECPs:

- Must hold a 4-year BHS degree in EMC.
- Must be currently registered as an ECP with the Health Professions Council of South Africa (HPCSA).

### Participant recruitment

Participant recruitment was conducted via strategically placed posters around campus and particularly outside the simulation laboratory on campus, enabling voluntary participation. Qualified ECPs who frequent the campus as tutors for the Department of Emergency Medical Care were also exposed to these posters on campus, inviting them to participate. Participants scanned a QR code, which led them to a website where they could provide their contact details to H.S. H.S. then contacted the potential participants via email or WhatsApp. Eligibility was confirmed before enrolment, and participants were anonymised through assigned identification numbers. Demographic data were collected confidentially.

### Assessment and evaluation

Assessments were conducted in a simulation laboratory designed to replicate an ED environment. A trained standardised patient actor presented the three clinical scenarios developed by the authors and therefore included input from a chiropractic and EMC perspective. Each participant had 30 min to complete assessments of all three cases, and all participants completed within this time allocation. The process involved reviewing paper-based case histories, performing clinical assessment and further history-taking, formulating a probable diagnosis, determining appropriate referrals and suggesting necessary further investigations.

Two independent assessors who were not part of the research study (an emergency care lecturer and a chiropractic lecturer) scored participants’ performances individually, using the simulation assessment tool for limiting assessor bias (SATLAB) system to reduce bias and enhance reliability. Assessments took place over 3 days.

The clinical cases are included in the supplementary information and include:

- *Case 1*: Musculoskeletal presentation; non-MSK emergency pathology (diagnosis: meningitis).
- *Case 2*: Musculoskeletal presentation; genuine MSK condition (diagnosis: intervertebral disc herniation).
- *Case 3*: Non-MSK presentation; non-MSK condition (diagnosis: stroke).

These cases were designed to evaluate diagnostic clinical assessment, diagnostic ability and appropriate referral skills.

Scoring employed the SATLAB assessment system, which evaluated three equally weighted outcomes (each contributing 33.33%):

- *Outcome 1*: Diagnostic assessment of the patient.
- *Outcome 2*: Diagnosis of the patient.
- *Outcome 3*: Clinical and diagnostic investigation referrals.

Each outcome was rated on a scale from 3 (best practice) to 0 (omitted). Management decisions were not evaluated, as this was not the aim of this study. Data were entered into Microsoft Excel for percentage calculations according to SATLAB guidelines (Makkink & Vincent Lambert 2020). The average between the two assessors was calculated for each outcome per participant. This was then multiplied by the 33.33% weighting, and the scores for the three outcomes were calculated. Data collection took place between 07 June 2024 and 10 July 2024.

### Assessment validity

The SATLAB tool has documented validity in a previous study (Makkink & Vincent-Lambert 2020). Case scenarios and the assessment rubric were developed by the authors and reviewed by three experienced ECPs familiar with SATLAB to ensure content validity.

### Data analysis

Statistical analysis was performed using IBM SPSS Statistics (version 29). The collected data underwent statistical analysis to evaluate performance differences among the three groups. Descriptive statistics, including means and percentages, were calculated to summarise demographic characteristics and performance scores across the three case scenarios and their constituent outcomes. For inferential analysis, a combination of parametric and non-parametric tests was employed to accommodate the study’s pilot nature and sample size. The Kruskal-Wallis test, a non-parametric alternative to a one-way analysis of variance (ANOVA), was used to identify any significant differences in scores among the three independent groups for each case and outcome. Upon finding significance, post-hoc analyses were conducted using Scheffé and Dunnett tests to pinpoint specific inter-group differences. Statistical significance was set at *p* ≤ 0.05.

### Ethical considerations

This study was approved by the University of Johannesburg, Faculty of Health Sciences, Research Ethics Committee (REC -2781-2024). Participants were provided with study information in an invitation email and in the study cover sheet. The study information described the purpose of the study, estimated time of completion, the voluntary and anonymous nature of participation, the right to withdraw from the study at any time without consequence, the confidentiality of collected data, and that the reporting of results would be in aggregate form. The independent assessors and the standardised patients signed confidentiality agreements to protect the identity of the participants. The identities of the participants were not divulged to I.B. or C.Y. because of the potential vulnerability of the registered students. H.S. managed the participants and the simulations.

## Results

A total of 30 participants completed the assessment over 3 days, with 73.33% (*n* = 22) female and 26.67% (*n* = 8) male participants. The age distribution was 43.33% (*n* = 13) aged 20–24 years, 40.00% (*n* = 12) aged 25–30 years, 13.33% (*n* = 4) aged 31–35 years, and 3.33% (*n* = 1) aged 36–40 years.

### Diagnosis

Regarding diagnostic accuracy (Table 1), 100% (*n* = 10) of first-year MHSc chiropractic students and ECPs correctly diagnosed Case 1 (meningitis), compared to 90.00% (*n* = 9) of second-year MHSc chiropractic students. For Case 2 (intervertebral disc herniation), 90.00% (*n* = 9) of both first- and second-year MHSc chiropractic students accurately identified the condition, while 70% (*n* = 7) of ECPs did so. In Case 3 (stroke), 100% (*n* = 10) of first-year MHSc chiropractic students and 100% (*n* = 9) of ECPs correctly diagnosed the condition, with 90.00% (*n* = 9) of second-year MHSc chiropractic students also achieving accurate diagnoses.

**TABLE 1:** Diagnostic accuracy of Master of Health Science chiropractic students and emergency care practitioners across the three case scenarios.

Groups	Case 1 – Meningitis (*n* = 10)		Case 2 – Intervertebral disc herniation (*n* = 10)		Case 3 – Stroke (*n* = 10)	
	*n*	%	*n*	%	*n*	%
First-year MHSc	10	100.00	9	90.00	10	100.00
Second-year MHSc	9	90.00	9	90.00	9	90.00
ECP	10	100.00	7	70.00	10	100.00

MHSc, Master of Health Science; ECPs, emergency care practitioners.

### Referral pathways and special investigations

As presented in Table 2, for Case 1 (Meningitis), 90.00% (*n* = 9) of both first- and second-year MHSc chiropractic students recommended a lumbar puncture, whereas 70.00% (*n* = 7) of ECPs did the same. Blood tests were favoured by 60.00% (*n* = 6) of first-year and 70.00% (*n* = 7) of second-year chiropractic students, compared with 90.00% (*n* = 9) of ECPs. Computed tomography (CT) scans were suggested by 40.00% (*n* = 4) of first-year, 70.00% (*n* = 7) of second-year MHSc chiropractic students, and 70.00% (*n* = 7) of ECPs. Magnetic resonance imaging (MRI) scans were recommended by 60% (*n* = 6) of first-year and 70.00% (*n* = 7) of second-year MHSc chiropractic students, and 40.00% (*n* = 4) of ECPs. Notably, none of the MHSc chiropractic students recommended serological tests, whereas 30% (*n* = 3) of the ECPs did. Additionally, 70.00% (*n* = 7) of all groups suggested a referral to a neurologist.

**TABLE 2:** Comparison of referral patterns and special investigations among Master of Health Science chiropractic students and emergency care practitioners in Case 1 – Meningitis.

Case 1 (*n* = 10)	Lumbar puncture		Blood tests		Computed tomography (CT)		Magnetic resonance imaging (MRI)		Serological tests		Referral to neurologist	
	*N*	%	*n*	%	*n*	%	*n*	%	*n*	%	*n*	%
First-year MHSc	9	90.00	6	60.00	4	40.00	6	60.00	0	0	7	70.00
Second-year MHSc	9	90.00	7	70.00	7	70.00	7	70.00	0	0	7	70.00
ECP	7	70.00	9	90.00	7	70.00	4	40.00	3	0	7	70.00

MHSc, Master of Health Science; ECPs, emergency care practitioners.

For Case 2 (Intervertebral Disc Herniation) presented in Table 3, 90.00% (*n* = 9) of first- and second-year chiropractic students recommended an MRI scan, whereas only 30.00% (*n* = 3) of ECPs did. Computed tomography scans were suggested by 20.00% (*n* = 2) of first-year and 30.00% (*n* = 3) of second-year MHSc chiropractic students, compared with 40.00% (*n* = 4) of ECPs. No participants recommended EMG/nerve conduction studies. Neurologist referrals were proposed by 40.00% (*n* = 4) of all groups, whereas orthopaedic referrals were suggested by 60.00% (*n* = 6) of first-year, 50.00% (*n* = 5) of second-year MHSc chiropractic students, and 70.00% (*n* = 7) of ECPs. Finally, referrals to chiropractors for further investigation were recommended by 50.00% (*n* = 5) of first-year, 30.00% (*n* = 3) of second-year MHSc chiropractic students, and 10.00% (*n* = 1) of ECPs.

**TABLE 3:** Comparison of referral patterns and special investigations among Master of Health Science chiropractic students and emergency care practitioners in Case 2 – Intervertebral disc herniation.

Case 2 (*n* = 10)	Magnetic resonance imaging (MRI)		Computed tomography (CT)		EMG/Nerve conduction studies		Neurologist		Orthopaedic surgeon		Chiropractor	
	*n*	%	*n*	%	*n*	%	*n*	%	*n*	%	*n*	%
First-year MHSc	9	90	2	20	0	0	4	40	6	60	5	50
Second-year MHSc	9	90	3	30	0	0	4	40	5	50	3	30
ECP	3	30	4	40	0	0	4	40	7	70	1	10

MHSc, Master of Health Science; ECPs, emergency care practitioners.

For Case 3 (stroke) and presented in Table 4, 90.00% (*n* = 9) of both first- and second-year MHSc chiropractic students recommended an MRI scan, whereas only 30.00% (*n* = 3) of ECPs did so. Computed tomography scans were suggested by 20% (*n* = 2) of first-year and 30.00% (*n* = 3) of second-year MHSc chiropractic students and by 40.00% (*n* = 4) of ECPs. Electromyography (EMG)/nerve conduction studies were not recommended by any participant. Referrals to a neurologist were suggested by 40.00% (*n* = 4) across all groups. Orthopaedic referrals were proposed by 60.00% (*n* = 6) of first-year students, 50.00% (*n* = 5) of second-year MHSc chiropractic students, and 70.00% (*n* = 7) of ECPs. Finally, referrals to chiropractors were recommended by 50.00% (*n* = 5) of first-year, 30.00% (*n* = 3) of second-year MHSc chiropractic students, and 10.00% (*n* = 1) of ECPs.

**TABLE 4:** Comparison of referral patterns and special investigations among Master of Health Science chiropractic students and emergency care practitioners in Case 3 – Stroke.

Case 3 (*n* = 10)	Magnetic resonance imaging (MRI)		Computed tomography (CT)		Intravenous thrombolytic therapy		Neurologist	
	*n*	%	*N*	%	*n*	%	*n*	%
First-year MHSc	4	40	5	50	0	0	6	60
Second-year MHSc	4	40	2	20	0	0	7	70
ECP	2		10		6		8	

MHSc, Master of Health Science; ECPs, emergency care practitioners.

### Mean scores

Performance across three simulated cases was compared between first-year MHSc students (*n* = 10), second-year MHSc students (*n* = 10) and BHS EMC graduates (*n* = 10). Parametric data were analysed using one-way ANOVA and non-parametric data using the Kruskal-Wallis H test. Post-hoc analyses (Scheffé and Dunnett) were conducted for significant ANOVA results. Descriptive and inferential statistics are presented in Table 5 (parametric data) and Table 6 (non-parametric data).

**TABLE 5:** Mean outcome scores and parametric results for Master of Health Science chiropractic students and emergency care practitioners across the three case scenarios.

Case	Groups compared and statistical test	*N*	Mean	s.d.	95% CI	
					Lower bound	Upper bound
Case 1: Outcome 1 – Score	First-years MHSc	10.00	54.20	24.02	37.02	71.38
	Second-year MHSc	10.00	68.40	22.67	52.18	84.62
	BHS EMC graduate	10.00	67.40	19.06	53.76	81.04
	Total	30.00	63.33	22.24	55.03	71.64
	ANOVA	*F*(2) = 1.29, *p* = 0.29	-	-	-	-
Case 1: Outcome 3 – Score	First-years MHSc	10.00	76.60	13.37	67.04	86.16
	Second-year MHSc	10.00	77.50	16.73	65.53	89.47
	BHS EMC graduate	10.00	76.60	10.20	69.30	83.90
	Total	30.00	76.90	13.22	71.96	81.84
	ANOVA	*F*(2) = 0.01, *p* = 0.99	-	-	-	-
Case 1: Final score	First-years MHSc	10.00	71.90	7.89	66.25	77.55
	Second-year MHSc	10.00	74.80	12.97	65.52	84.08
	BHS EMC graduate	10.00	79.10	9.41	72.37	85.83
	Total	30.00	75.27	10.40	71.38	79.15
	ANOVA	*F*(2) = 1.23, *p* = 0.31	-	-	-	-
Case 2: Outcome 1 – Score	First-years MHSc	10.00	64.90	26.19	46.16	83.64
	Second-year MHSc	10.00	70.10	33.01	46.49	93.71
	BHS EMC graduate	10.00	59.20	16.09	47.69	70.71
	Total	30.00	64.73	25.53	55.20	74.27
	ANOVA	*F*(2) = 0.44, *p* = 0.65	-	-	-	-
Case 2: Outcome 3 – Score	First-years MHSc	10.00	77.50	19.20	63.76	91.24
	Second-year MHSc	10.00	89.20	16.25	77.58	100.82
	BHS EMC graduate	10.00	52.40	13.18	42.97	61.83
	Total	30.00	73.03	22.23	64.73	81.33
	ANOVA	*F*(2) = 13.15, *p* < 0.01	-	-	-	-
Post-hoc	Scheffe and Dunnett	*p* < 0.01	-	-	-	-
Case 2: Final score	First-years MHSc	10.00	75.20	15.12	64.38	86.02
	Second-year MHSc	10.00	83.00	20.10	68.62	97.38
	BHS EMC graduate	10.00	59.10	12.22	50.36	67.84
	Total	30.00	72.43	18.58	65.50	79.37
	ANOVA	*F*(2) = 5.70, *p* < 0.01	-	-	-	-
Post-hoc	Scheffe and Dunnett	*p* = 0.01 (2nd year MHSc and ECP)	-	-	-	-
Case 3: Outcome 1 – Score	First-years MHSc	10.00	57.60	31.52	35.05	80.15
	Second-year MHSc	10.00	46.80	26.94	27.53	66.07
	BHS EMC graduate	10.00	63.40	14.68	52.90	73.90
	Total	30.00	55.93	25.48	46.42	65.45
	ANOVA	*F*(2) = 1.10, *p* = 0.35	-	-	-	-
Case 3: Outcome 3 – Score	First-years MHSc	10.00	69.20	20.50	54.53	83.87
	Second-year MHSc	10.00	72.50	22.96	56.07	88.93
	BHS EMC graduate	10.00	81.80	12.25	73.04	90.56
	Total	30.00	74.50	19.24	67.32	81.68
	ANOVA	*F*(2) = 1.17, *p* = 0.33	-	-	-	-
Case 3: Final score	First-years MHSc	10.00	72.10	14.50	61.73	82.47
	Second-year MHSc	10.00	67.40	16.41	55.66	79.14
	BHS EMC graduate	10,00	79.50	11.28	71.43	87.57
	Total	30.00	73.00	14.63	67.54	78.46
	ANOVA	*F*(2) = 1.84, *p* = 0.18	-	-	-	-

MHSc, Master of Health Science; ECPs, emergency care practitioners; BHS, Bachelor of Health Sciences; EMC, Emergency Medical Care; ANOVA, analysis of variance; CI, confidence interval; s.d., standard deviation.

**TABLE 6:** Mean ranks, outcome scores and non-parametric results for Master of Health Science chiropractic students and emergency care practitioners across the three case scenarios.

Case	Groups compared and statistical test	*N*	Mean rank
Case 1: Outcome 2 – Score	First-years MHSc	10.00	12.90
	Second-year MHSc	10.00	13.70
	BHS EMC graduate	10.00	19.90
	Total	30.00	-
	Kruskal-Wallis	χ^2^(2) = 5.07, *p* = 0.08	-
Case 2: Outcome 2 – Score	First-years MHSc	10.00	15.90
	Second-year MHSc	10.00	21.55
	BHS EMC graduate	10.00	9.05
	Total	30.00	-
	Kruskal-Wallis	χ^2^(2) = 11.08, *p* < 0.01	-
Case 3: Outcome 2 – Score	First-years MHSc	10.00	14.80
	Second-year MHSc	10.00	14.00
	BHS EMC graduate	10.00	17.70
	Total	30.00	-
Kruskal-Wallis	χ^2^(2) = 1.26, *p* = 0.53	-

MHSc, Master of Health Science; BHS, Bachelor of Health Sciences; EMC, Emergency Medical Care.

#### Case 1

No statistically significant differences were found between the groups for Outcome 1 (*F*(2) = 1.29, *p* = 0.29), Outcome 3 (*F*(2) = 0.01, *p* = 0.99) or the Case 1 Final Score (*F*(2) = 1.23, *p* = 0.31). Similarly, the Kruskal-Wallis test revealed no significant difference in scores for Outcome 2 (χ^2^(2) = 5.07, *p* = 0.08).

#### Case 2

For Outcome 1, no significant difference between groups was found (*F*(2) = 0.44, *p* = 0.65). A significant difference was identified for Outcome 2 (χ^2^(2) = 11.08, *p* < 0.01). The mean ranks indicate that second-year MHSc students (Mean Rank = 21.55) scored the highest, followed by first-year students (Mean Rank = 15.90), with BHS EMC graduates (Mean Rank = 9.05) scoring the lowest.

A one-way ANOVA showed a significant effect of group on Outcome 3 scores (*F*(2) = 13.15, *p* < 0.01). Post-hoc analyses (Scheffé and Dunnett) confirmed that BHS EMC graduates (*M* = 52.40, s.d. = 13.18) scored significantly lower than both first-year (*M* = 77.50, s.d. = 19.20) and second-year MHSc students (*M* = 89.20, s.d. = 16.25) (*p* < 0.01).

This effect was also reflected in the Case 2 Final Score, where a one-way ANOVA was significant (*F*(2) = 5.70, *p* < 0.01). Post-hoc tests specified that second-year MHSc students (*M* = 83.00, s.d. = 20.10) achieved a significantly higher final score than BHS EMC graduates (*M* = 59.10, s.d. = 12.22) (*p* = 0.01).

#### Case 3

No statistically significant differences were found between the groups for Outcome 1 (*F*(2) = 1.10, *p* = 0.35), Outcome 3 (*F*(2) = 1.17, *p* = 0.33) or the Case 3 Final Score (*F*(2) = 1.84, *p* = 0.18). The Kruskal-Wallis test for Outcome 2 also showed no significant difference (χ^2^(2) = 1.26, *p* = 0.53).

## Discussion

This study explored the diagnostic capabilities of MHSc chiropractic students and ECPs in differentiating MSK from emergency pathologies in a simulated setting. The primary finding was that while performance was similar across groups for meningitis and stroke cases (Cases 1 and 3), second-year chiropractic students significantly outperformed ECPs in the clinical management of a lumbar disc herniation (Case 2).

No significant differences were observed between the three cohorts in Case 1 (Meningitis) and Case 3 (Stroke). However, Case 2 (Intervertebral Disc Herniation) revealed notable differences, especially in Outcomes 2 and 3 and the overall scores. While ECPs performed well in non-MSK cases, they demonstrated lower accuracy in diagnosing disc herniations compared to chiropractic students. Specifically, many ECPs frequently used the colloquial term ‘slipped disc’, suggesting potential gaps in understanding this pathology.

In contrast, first- and second-year MHSc chiropractic students exhibited superior diagnostic accuracy and more appropriate clinical decision-making, likely reflecting their focused training in MSK and neurological examinations. Magnetic resonance imaging is considered the appropriate imaging modality for disc herniations, with X-rays having limited benefit for such soft tissue pathology (Mai 2018; Van den Wyngaert 2024). Notably, 90% of second-year chiropractic students recommended MRI as the appropriate imaging modality for disc herniation, while only 30% of EMC graduates did the same and suggested X-rays and referrals to orthopaedic specialists. This pattern may reflect differences in curricular emphasis, with chiropractic programmes offering deeper coverage of diagnostic imaging for MSK conditions. The limited use of conservative referrals (e.g. to chiropractors) among ECPs highlights an area for potential improvement in their referral network, as, over and above referral to the hospital, they may also refer to additional appropriate health care providers. Although ECPs do not directly refer to other healthcare services (such as radiographic imaging), they do make decisions pertaining to the best healthcare facility to take patients to, thus requiring insight into appropriate investigations per pathology.

Existing literature highlights the global financial burden of lower back pain and the importance of accurate diagnosis and management of intervertebral disc herniations (Al Qaraghli & De Jesus 2021; Yang et al. 2015). While the ECPs’ preference was for orthopaedic referrals, which may not compromise patient safety, it could lead to unnecessary healthcare costs. Conversely, the chiropractic students’ stronger performance in this scenario suggests a potential role for them in improving cost-effective management of MSK complaints in emergency settings.

Importantly, while ECPs generally performed better in non-MSK cases, their performance was not significantly higher than that of the MHSc chiropractic students. This suggests that MHSc chiropractic students possess the foundational skills necessary to differentiate between emergency and MSK pathologies, reinforcing their potential contribution as primary care providers in EDs. Their ability to identify and manage MSK conditions accurately may help reduce ED workloads and facilitate more efficient patient care, without compromising patient safety through misdiagnosis of emergency pathologies.

Interestingly, performance varied between the first- and second-year MHSc chiropractic groups. The second-year group excelled in Cases 1 and 2, while the first-year group performed better in Case 3. These variations highlight possible influences such as case complexity, differences in educational emphasis per year of study, or group characteristics, which may warrant further investigation.

Given the established efficacy of physiotherapy-led services for MSK conditions within EDs internationally (Cassar et al. 2022; Gagnon et al. 2021; Harding et al. 2015; Matifat et al. 2023; Taylor et al. 2011), the preliminary findings of this study contribute to a growing body of evidence suggesting chiropractors could function effectively as primary contact practitioners in similar environments. For a country like South Africa, which faces a significant burden of MSK-related ED presentations (Parker & Jelsma 2010; Taljaard et al. 2022), future healthcare planning could explore the inclusion of chiropractors in multidisciplinary, conservative management programmes.

### Limitations

This study has several limitations that should be acknowledged. The selection of 10 participants per cohort limits the statistical power and generalisability of the findings. The focus on participants exclusively from the University of Johannesburg further constrains the applicability of the results to other institutions within South Africa or internationally. Additionally, the study was limited to MHSc chiropractic students, and the findings may not extend to qualified practitioners. The scope of the assessment was restricted to three specific clinical cases, which may not fully capture the breadth of diagnostic scenarios encountered in emergency settings. The simulated environment, while controlled, cannot replicate the pressures and complexities of an actual ED, potentially affecting the external validity of the results.

### Recommendations

Building on this study, several recommendations emerge for future research. A larger-scale studies are needed to enhance the representativeness and reliability of the findings, providing a more comprehensive understanding of diagnostic competencies among chiropractic students and ECPs. Including participants from both the University of Johannesburg and the Durban University of Technology would broaden the sample base and facilitate comparisons between South Africa’s chiropractic institutions.

Future research should also compare the performance of qualified, practising chiropractors with ECPs to assess clinical competency across levels of training and professional practice. Expanding the comparative framework to include other healthcare providers would further clarify the potential role of chiropractors within the multidisciplinary healthcare system.

## Conclusion

This study demonstrates that diagnostic and management capabilities are highly scenario-dependent. While ECPs demonstrated consistent strength in diagnosing non-MSK emergencies, second-year chiropractic students outperformed them in the comprehensive clinical management of an MSK condition (lumbar disc herniation). This superior performance, with more appropriate imaging recommendations and higher clinical reasoning scores, underscores focused MSK training within the chiropractic programme.

Importantly, chiropractic students performed on par with ECPs in identifying serious pathologies like meningitis and stroke, indicating they possess the necessary foundational competency to safely differentiate between MSK and emergency conditions.

These preliminary findings suggest that chiropractors, by virtue of their training, could be valuable assets in multidisciplinary teams addressing the high burden of MSK presentations in emergency settings, potentially improving cost-efficiency and patient flow. However, these results are from a small-scale simulation and require validation through larger, real-world studies. Future research should focus on implementing and evaluating such models, particularly in resource-constrained environments like South Africa.

## References

[CIT0001] Al Qaraghli, M. & De Jesus, O., 2021, *Lumbar disc herniation*, StatPearls Publishing, Treasure Island, FL. 32809713

[CIT0002] Cassar, M.R., Davies, F., Braddock, S., Massalha, V. & Pace, J., 2022, ‘The role for Physiotherapists in the management of minor musculoskeletal injuries presenting to an Emergency Department. An evaluation of the Physiotherapy service at the Emergency Department of Mater Dei Hospital’, *Malta Medical Journal* 34(2), 78–86, viewed 05 September 2025, from https://www.scopus.com/inward/record.uri?eid=2-s2.0-85130615977&partnerID=40&md5=5056f41884b9f8e341fdef7441f4aedc.

[CIT0003] Comer, G.C., Liang, E. & Bishop, J.A., 2014, ‘Lack of proficiency in musculoskeletal medicine among emergency medicine physicians’, *Journal of Orthopaedic Trauma* 28(4), e85–e87. 10.1097/bot.0b013e3182a66829 23899765

[CIT0004] Denq, W., Tomesch, A.J., Lane, A.D., Thomas, A., Mcninch, N.L. & Waterbrook, A., 2023, ‘National needs assessment of emergency medicine residencies for musculoskeletal knowledge’, *Cureus* 15(8), e43638. 10.7759/cureus.43638 37719484 PMC10504909

[CIT0005] Fialho, S.C.M.S., De Castro, G.R.W., Zimmermann, A.F., Ribeiro, G.G., Neves, F.S., Pereira, I.A. et al., 2011, ‘Musculoskeletal system assessment in an emergency room’, *Revista Brasileira de Reumatologia* 51(3), 240–248. 10.1590/S0482-50042011000300005 21625812

[CIT0006] Gagnon, R., Perreault, K., Berthelot, S., Matifat, E., Desmeules, F., Achou, B. et al., 2021, ‘Direct-access physiotherapy to help manage patients with musculoskeletal disorders in an emergency department: Results of a randomized controlled trial’, *Academic Emergency Medicine* 28(8), 848–858. 10.1111/acem.14237 33617696

[CIT0007] Gagnon, R., Perreault, K., Guertin, J.R., Berthelot, S., Achou, B. & Hébert, L.J., 2022, ‘Health-related quality of life of patients presenting to the emergency department with a musculoskeletal disorder’, *ClinicoEconomics and Outcomes Research* 14, 91–103. 10.2147/ceor.s348138 35221700 PMC8865860

[CIT0008] Haldeman, S., Nordin, M., Outerbridge, G., Hurwitz, E.L., Hondras, M., Brady, O.D. et al., 2015, ‘Creating a sustainable model of spine care in underserved communities: The World Spine Care (WSC) charity’, *The Spine Journal* 15(11), 2303–2311. 10.1016/j.spinee.2015.06.046 26096472

[CIT0009] Harding, P., Prescott, J., Block, L., O’flynn, A.M. & Burge, A.T., 2015, ‘Patient experience of expanded-scope-of-practice musculoskeletal physiotherapy in the emergency department: A qualitative study’, *Australian Health Review* 39(3), 283–289. 10.1071/ah14207 25913520

[CIT0010] Ingram, M. & Symmons, D.P.M., 2018, ‘The burden of musculoskeletal conditions’, *Medicine* 46(3), 152–155. 10.1016/j.mpmed.2017.12.005

[CIT0011] Joshipura, M. & Gosselin, R.A., 2020, ‘Surgical burden of musculoskeletal conditions in low- and middle-income countries’, *World Journal of Surgery* 44(4), 1026–1032. 10.1007/s00268-018-4790-8 30238386

[CIT0012] Kindermann, S.L., Hou, Q. & Miller, R.M., 2014, ‘Impact of chiropractic services at an on-site health center’, *Journal of Occupational and Environmental Medicine* 56(9), 990–992. 10.1097/jom.0000000000000215 25153305

[CIT0013] Lisi, A.J., Salsbury, S.A., Twist, E.J. & Goertz, C.M., 2018, ‘Chiropractic integration into private sector medical facilities: A multisite qualitative case study’, *The Journal of Alternative and Complementary Medicine* 24(8), 792–800. 10.1089/acm.2018.0218 30016118 PMC6909703

[CIT0014] Louw, Q.A., Morris, L.D. & Grimmer-Somers, K., 2007, ‘The prevalence of low back pain in Africa: A systematic review’, *BMC Musculoskeletal Disorders* 8, 105. 10.1186/1471-2474-8-105 17976240 PMC2198912

[CIT0015] Lowe, D.B., Taylor, M.J. & Hill, S.J., 2017, ‘Associations between multimorbidity and additional burden for working-age adults with specific forms of musculoskeletal conditions: A cross-sectional study’, *BMC Musculoskeletal Disorders* 18(1), 135. 10.1186/s12891-017-1496-2 28376838 PMC5379740

[CIT0016] Mai, W, 2018, ‘Normal MRI spinal anatomy, degenerative disc disease, and disc herniation’, in W. Mai (ed.), *Diagnostic MRI in dogs and cats*, pp. 412–416, CRC Press, Florida.

[CIT0017] Makkink, A.W. & Vincent-Lambert, C., 2020, ‘The development of “SATLAB”: A tool designed to limit assessment bias in simulation-based learning’, *South African Journal of Pre-hospital Emergency Care* 1(1), 3024. 10.24213/1-1-3024

[CIT0018] Matifat, E., Berger Pelletier, E., Brison, R., Hébert, L.J., Roy, J.S., Woodhouse, L. et al., 2023, ‘Advanced practice physiotherapy care in emergency departments for patients with musculoskeletal disorders: A pragmatic cluster randomized controlled trial and cost analysis’, *Trials* 24(1), 84. 10.1186/s13063-023-07100-x 36747305 PMC9900999

[CIT0019] Menke, J.M., 2003, ‘Principles in integrative chiropractic’, *Journal of Manipulative and Physiological Therapeutics* 26(4), 254–272. 10.1016/S0161-4754(02)54113-0 12750661

[CIT0020] Murphy, D.R., Justice, B.D., Paskowski, I.C., Perle, S.M. & Schneider, M.J., 2011, ‘The establishment of a primary spine care practitioner and its benefits to health care reform in the United States’, *Chiropractic & Manual Therapies* 19(1), 17. 10.1186/2045-709x-19-17 21777444 PMC3154851

[CIT0021] Parker, R. & Jelsma, J., 2010, ‘The prevalence and functional impact of musculoskeletal conditions amongst clients of a primary health care facility in an under-resourced area of Cape Town’, *BMC Musculoskeletal Disorders* 11, 2. 10.1186/1471-2474-11-2 20044944 PMC2830178

[CIT0022] Pasta, G., Polizzi, A., Annunziata, S., Klersy, C., Fenech, L., Farahani, M.R.D. et al., 2022, ‘Patients with musculoskeletal disorders presenting to the Emergency Department: The COVID-19 lesson’, *International Journal of Environmental Research and Public Health* 19(10), 5891. 10.3390/ijerph19105891 35627428 PMC9140523

[CIT0023] Suter, E., Boakye, O., Birney, A., Phillips, L. & Suen, V., 2016, ‘Scope of practice review: Providers for triage and assessment of spine-related disorders’, *Journal of Multidisciplinary Healthcare* 9, 227–235. 10.2147/jmdh.s97590 27274267 PMC4869847

[CIT0024] Taljaard, L., Maharaj, R. & Hendrikse, C., 2022, ‘A descriptive analysis of the casemix presenting to a tertiary hospital emergency centre in East London, South Africa’, *African Journal of Emergency Medicine* 12(3), 252–258. 10.1016/j.afjem.2022.05.006 35795815 PMC9249588

[CIT0025] Taylor, N.F., Norman, E., Roddy, L., Tang, C., Pagram, A. & Hearn, K., 2011, ‘Primary contact physiotherapy in emergency departments can reduce length of stay for patients with peripheral musculoskeletal injuries compared with secondary contact physiotherapy: A prospective non-randomised controlled trial’, *Physiotherapy* 97(2), 107–114. 10.1016/j.physio.2010.08.011 21497244

[CIT0026] University of Southern Denmark, 2023, *The international chiropractic education collaboration*, viewed 28 May 2025, from https://www.sdu.dk/en/icec.

[CIT0027] Van den Wyngaert, T., 2024, ‘Degenerative spine: Disc herniation’, in T. Van den Wyngaert, G. Gnanasegaran & K. Strobel (eds.), *Clinical atlas of bone SPECT/CT*, pp. 87–90, Springer Nature, Cham.

[CIT0028] Weinstein, S.L., 2016, ‘The burden of musculoskeletal conditions’, *The Journal of Bone and Joint Surgery* 98(16), 1331. 10.2106/jbjs.16.00595 27535434

[CIT0029] Woolf, A.D. & Åkesson, K., 2001, ‘Understanding the burden of musculoskeletal conditions’, *BMJ* 322(7294), 1079–1080. 10.1136/bmj.322.7294.1079 11337425 PMC1120225

[CIT0030] Yang, H., Liu, H., Li, Z., Zhang, K., Wang, J., Wang, H. et al., 2015, ‘Low back pain associated with lumbar disc herniation: Role of moderately degenerative disc and annulus fibrous tears’, *International Journal of Clinical and Experimental Medicine* 8(2), 1634–1644. 25932092 PMC4402739

